# Somatic mutations and increased lymphangiogenesis observed in a rare case of intramucosal gastric carcinoma with lymph node metastasis

**DOI:** 10.18632/oncotarget.24289

**Published:** 2018-01-22

**Authors:** Naoki Ikari, Shota Aoyama, Akiyoshi Seshimo, Yuji Suehiro, Tomoko Motohashi, Shohei Mitani, Sawako Yoshina, Etsuko Tanji, Akiko Serizawa, Takuji Yamada, Kiyoaki Taniguchi, Masakazu Yamamoto, Toru Furukawa

**Affiliations:** ^1^ Department of Surgery, Institute of Gastroenterology, Tokyo Women's Medical University, Tokyo, Japan; ^2^ Institute for Integrated Medical Sciences, Tokyo Women's Medical University, Tokyo, Japan; ^3^ Department of Physiology, Tokyo Women's Medical University School of Medicine, Tokyo, Japan; ^4^ Department of Surgical Pathology, Tokyo Women's Medical University Hospital, Tokyo, Japan; ^5^ Department of Histopathology, Tohoku University Graduate School of Medicine, Sendai, Japan

**Keywords:** early gastric cancer, lymph node metastasis, lymphangiogenesis, *NBN*, *PAX8*

## Abstract

**Background and aim:**

Intramucosal gastric adenocarcinoma of the well-moderately differentiated type only exhibits lymph node metastasis in extremely rare cases. We encountered such case and investigated both the lymphangiogenic properties and somatic mutations in the cancer to understand the prometastatic features of early-stage gastric cancer.

**Methods:**

We quantitatively measured the density of lymphatic vessels and identified mutations in 412 cancer-associated genes through next-generation target resequencing of DNA extracted from tumor cells in a formalin-fixed and paraffin-embedded tissue. Functional consequence of the identified mutation was examined *in vitro* by means of gene transfection, immunoblot, and the quantitative real-time polymerase chain reaction assay.

**Results:**

The intramucosal carcinoma was accompanied by abundant lymphatic vessels. The metastatic tumor harbored somatic mutations in *NBN*, p.P6S, and *PAX8*, p.R49H. The *PAX8*^R49H^ showed significantly higher transactivation activity toward *E2F1* than the wild-type *PAX8* (P< 0.001).

**Conclusions:**

Our data suggest that increased lymphangiogenesis and somatic mutations of *NBN* and/or *PAX8* could facilitate lymph node metastasis from an intramucosal gastric carcinoma. These findings may potentially inform evaluations of the risk of developing lymph node metastasis in patients with intramucosal gastric cancer.

## INTRODUCTION

An endoscopic resection is recommended as a standard treatment (absolute indication) for early gastric carcinomas that fulfill the following criteria: a differentiated-type adenocarcinoma without ulcerative findings, of which the depth of invasion is clinically diagnosed as cT1a and the diameter is ≤ 2 cm [1]. This recommendation is based on the rare occurrence of lymph node (LN) metastasis, which is reported to be 0.12% (3/2402) or 0% (0/6456), in patients with early gastric cancer fulfilling the above criteria [1–3]. Despite the rare occurrence of LN metastasis, published reports have emphasized the importance of careful evaluation of LN state by using computed tomography (CT) because if a LN metastasis is found, a surgical resection with lymphadenectomy should be performed instead of the endoscopic resection [2, 4]. Hence, mechanistic insights into LN metastasis from early gastric cancer could provide clues for improving the present criteria for the absolute indication of endoscopic resection for early gastric cancer. We obtained a rare opportunity to explore a case of intramucosal gastric adenocarcinoma with synchronized LN metastasis. In this case, we performed a quantitative lymphatic vessel density evaluation, targeted resequencing of 412 cancer-associated genes by next-generation sequencing technology, and a subsequent functional analysis for a mutated gene.

## RESULTS

### Patient characteristics and clinical course

**Figure 1:**
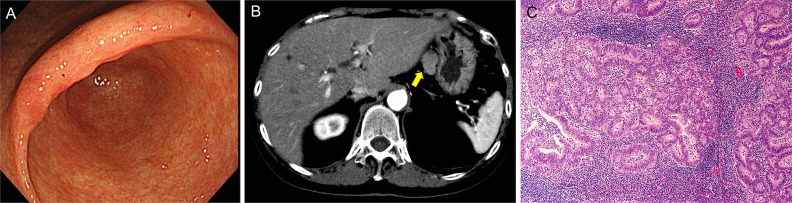
Preoperative findings **(A)** Endoscopy showed irregular mucosa in the lesser curvature of the gastric angle with ill-defined margins from surrounding atrophic mucosa. **(B)** Computed tomography showed an irregular oval tumor of 23 mm diameter beside the left gastric artery (arrow). **(C)** Laparoscopic biopsy of the intra-abdominal tumor revealed a metastatic tubular adenocarcinoma in the lymph node. Hematoxylin and eosin (H&E) staining, original magnification, ×100.

A 68-year-old woman, with a history of eradicated *Helicobacter pylori* infection, suffered from transient epigastralgia. The patient underwent upper gastrointestinal endoscopy, which elucidated an irregular mucosal lesion in the gastric angle (Figure 1A). A biopsy revealed a tubular adenocarcinoma of the well-differentiated type. Carcinoembryonic antigen (CEA) and carbohydrate antigen 19-9 (CA19-9) levels were 8.5 ng/ml and 50 U/ml, respectively. Although the lesion seemed to be confined within the gastric mucosa and to fulfill the absolute indication of endoscopic resection, a suspicious LN metastatic lesion (23 mm diameter) beside the left gastric artery was noted on a CT scan (Figure 1B). A laparoscopic tumor biopsy revealed that the tumor was an enlarged LN with tubular adenocarcinoma that was histologically similar to the gastric tumor (Figure 1C). Positron emission tomography/CT showed no apparent uptake except in the LN tumor. Thus, the patient was diagnosed to have an early gastric cancer with a regional LN metastasis and underwent the standard distal gastrectomy with D2 lymph node dissection. After surgery, CEA and CA19-9 levels normalized. The patient underwent no adjuvant chemotherapy and has remained in good health without any signs of recurrence or other malignant tumors for 39 months (most recent follow-up).

### Pathological findings

**Figure 2:**
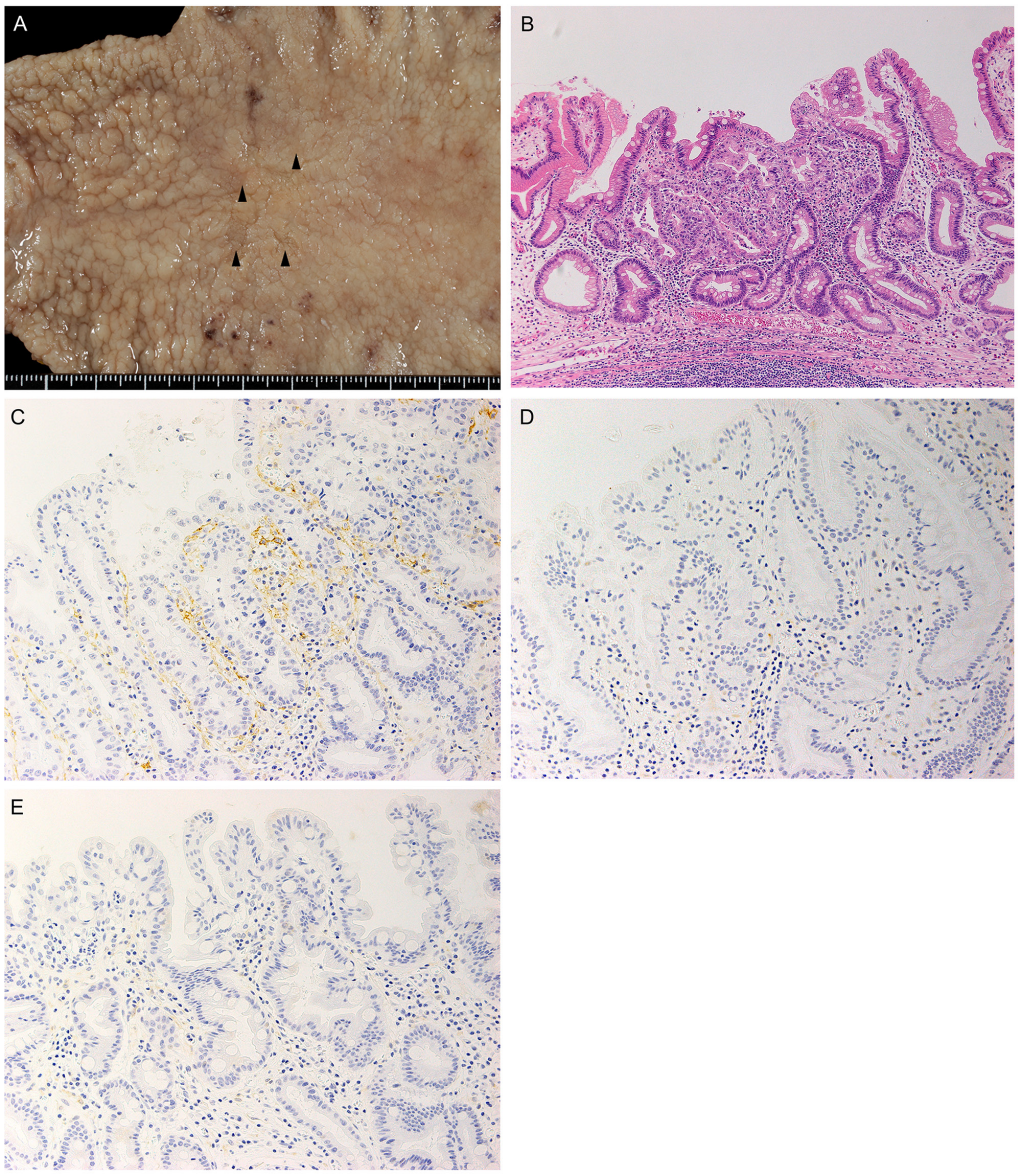
Gross and microscopic pathology of the surgically resected section of the stomach **(A)** Four irregular mucosal lesions, each 1-4 mm in diameter, were located side by side in the lesser curvature (arrowheads). **(B)** The primary cancer consisted of tubular adenocarcinoma of the well-differentiated type confined within the mucosal layer without ulceration. H&E staining, original magnification, ×100. **(C-E)** Images of immunohistochemical staining with anti-D2-40 antibody showed abundant lymphatic vessels in an intratumoral area (C) in contrast with scanty lymphatic vessels in a peritumoral area (D) and a portion of normal mucosa (E). All histological images were taken at the original magnification of ×200.

A pathological examination identified 4 independent lesions, 1–4 mm in diameter, in close vicinity to each other at the gastric angle (Figure 2A). The lesions consisted of well-differentiated tubular adenocarcinomas confined within the mucosal layer without any apparent ulceration (Figure 2B, Supplementary Figure 1). There was no scar formation that could suggest segregation of an originally existing tumor into 4 cancerous lesions. The histological features were identical to those of the metastatic adenocarcinoma in the LN along the lesser curvature (Figure 1C, 2B).

### Evaluation of lymphatic vessels

Formalin-fixed and paraffin-embedded (FFPE) tissue sections were immunohistochemically stained with anti-D2-40 antibody. D2-40-positive lymphatic vessels were particularly dense in the lamina propria of the intramucosal tubular adenocarcinoma in the primary tumor. The dense lymphatic vessels showed irregular shapes with collapsing compared to those in normal mucosa (Figure 2C–2E). Lymphatic vessel density was evaluated semi-quantitatively as described by Pak et al [5]. Intratumoral lymphatic vessel density (I-LVD), peritumoral lymphatic vessel density (P-LVD), and control lymphatic vessel density (C-LVD) were 72.3 ± 4.5, 10.7 ± 3.8, and 10.7 ± 8.3, respectively. The I-LVD was significantly higher than P-LVD (P< 0.001) and C-LVD (P< 0.001). Moreover, I-LVD in this patient was strikingly higher than the mean values reported by Pak et al. for node-negative cases, 11.26± 3.84, and N3 cases, 14.16 ± 5.00 [5].

### Somatic mutations

**Figure 3:**
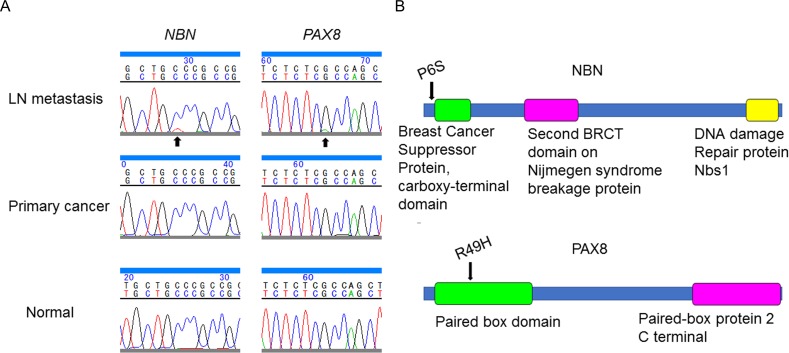
Somatic mutations in *NBN* and *PAX8* identified in the present case **(A)** Validation by Sanger sequencing showed single nucleotide substitution in *NBN* and *PAX8* in the lymph node metastasis (arrow) but not in the primary tumor. **(B)** Functional domains (colored boxes) and mutated residues (arrows) in nibrin (NBN) and paired box 8 (PAX8).

We prepared DNA from microdissected FFPE tissue samples of the primary intramucosal gastric carcinoma, the metastatic LN carcinoma, and a normal tissue, and performed target resequencing using an Ion Proton System (Thermo Fisher Scientific, Carlsbad, CA, USA). We employed two panels of target genes to cover the known commonly mutated genes in gastric cancer (Table 1) [6–8]. One panel was the IonAmpliSeq™ Comprehensive Cancer Panel (Thermo Fisher Scientific) that covered the coding exons of 409 cancer-associated genes, and the other was an Ion AmpliSeq™ Custom DNA Panel (Thermo Fisher Scientific) that was designed to cover the coding exons of *RHOA* and its regulatory molecules, *AKAP13* and *DLC1* (Table 1). The mean coverage of each panel was 241.4 and 4816.3 fold per amplicon, respectively. We identified somatic mutations in *NBN,* p.P6S, and *PAX8,* p.R49H, in the LN metastasis; however, we did not identify these mutations in the primary tumor (Table 2). These mutations were confirmed by Sanger sequencing (Figure 3A, 3B).

**Table 1:** The list of target genes examined in panel sequencing

*ABL1*	*CASC5*	*EGFR*	*G6PD*	*KLF6*	*MYCN*	*PIM1*	*SH2D1A*	*USP9X*
*ABL2*	*CBL*	*EML4*	*GATA1*	*KRAS*	*MYD88*	*PKHD1*	*SMAD2*	*VHL*
*ACVR2A*	*CCND1*	*EP300*	*GATA2*	*LAMP1*	*MYH11*	*PLAG1*	*SMAD4*	*WAS*
*ADAMTS20*	*CCND2*	*EP400*	*GATA3*	*LCK*	*MYH9*	*PLCG1*	*SMARCA4*	*WHSC1*
*AFF1*	*CCNE1*	*EPHA3*	*GDNF*	*LIFR*	*NBN*	*PLEKHG5*	*SMARCB1*	*WRN*
*AFF3*	*CD79A*	*EPHA7*	*GNA11*	*LPHN3*	*NCOA1*	*PML*	*SMO*	*WT1*
*AKAP9*	*CD79B*	*EPHB1*	*GNAQ*	*LPP*	*NCOA2*	*PMS1*	*SMUG1*	*XPA*
*AKAP13*	*CDC73*	*EPHB4*	*GNAS*	*LRP1B*	*NCOA4*	*PMS2*	*SOCS1*	*XPC*
*AKT1*	*CDH1*	*EPHB6*	*GPR124*	*LTF*	*NF1*	*POT1*	*SOX11*	*XPO1*
*AKT2*	*CDH11*	*ERBB2*	*GRM8*	*LTK*	*NF2*	*POU5F1*	*SOX2*	*XRCC2*
*AKT3*	*CDH2*	*ERBB3*	*GUCY1A2*	*MAF*	*NFE2L2*	*PPARG*	*SRC*	*ZNF384*
*ALK*	*CDH20*	*ERBB4*	*HCAR1*	*MAFB*	*NFKB1*	*PPP2R1A*	*SSX1*	*ZNF521*
*APC*	*CDH5*	*ERCC1*	*HIF1A*	*MAGEA1*	*NFKB2*	*PRDM1*	*STK11*	
*AR*	*CDK12*	*ERCC2*	*HLF*	*MAGI1*	*NIN*	*PRKAR1A*	*STK36*	
*ARID1A*	*CDK4*	*ERCC3*	*HNF1A*	*MALT1*	*NKX2-1*	*PRKDC*	*SUFU*	
*ARID2*	*CDK6*	*ERCC4*	*HOOK3*	*MAML2*	*NLRP1*	*PSIP1*	*SYK*	
*ARNT*	*CDK8*	*ERCC5*	*HRAS*	*MAP2K1*	*NOTCH1*	*PTCH1*	*SYNE1*	
*ASXL1*	*CDKN2A*	*ERG*	*HSP90AA1*	*MAP2K2*	*NOTCH2*	*PTEN*	*TAF1*	
*ATF1*	*CDKN2B*	*ESR1*	*HSP90AB1*	*MAP2K4*	*NOTCH4*	*PTGS2*	*TAF1L*	
*ATM*	*CDKN2C*	*ETS1*	*ICK*	*MAP3K7*	*NPM1*	*PTPN11*	*TAL1*	
*ATR*	*CEBPA*	*ETV1*	*IDH1*	*MAPK1*	*NRAS*	*PTPRD*	*TBX22*	
*ATRX*	*CHEK1*	*ETV4*	*IDH2*	*MAPK8*	*NSD1*	*PTPRT*	*TCF12*	
*AURKA*	*CHEK2*	*EXT1*	*IGF1R*	*MARK1*	*NTRK1*	*RAD50*	*TCF3*	
*AURKB*	*CIC*	*EXT2*	*IGF2*	*MARK4*	*NTRK3*	*RAF1*	*TCF7L1*	
*AURKC*	*CKS1B*	*EZH2*	*IGF2R*	*MBD1*	*NUMA1*	*RALGDS*	*TCF7L2*	
*AXL*	*CMPK1*	*FAM123B*	*IKBKB*	*MCL1*	*NUP214*	*RARA*	*TCL1A*	
*BAI3*	*COL1A1*	*FANCA*	*IKBKE*	*MDM2*	*NUP98*	*RB1*	*TET1*	
*BAP1*	*CRBN*	*FANCC*	*IKZF1*	*MDM4*	*PAK3*	*RECQL4*	*TET2*	
*BCL10*	*CREB1*	*FANCD2*	*IL2*	*MEN1*	*PALB2*	*REL*	*TFE3*	
*BCL11A*	*CREBBP*	*FANCF*	*IL21R*	*MET*	*PARP1*	*RET*	*TGFBR2*	
*BCL11B*	*CRKL*	*FANCG*	*IL6ST*	*MITF*	*PAX3*	*RHOA*	*TGM7*	
*BCL2*	*CRTC1*	*FAS*	*IL7R*	*MLH1*	*PAX5*	*RHOH*	*THBS1*	
*BCL2L1*	*CSF1R*	*FBXW7*	*ING4*	*MLL*	*PAX7*	*RNASEL*	*TIMP3*	
*BCL2L2*	*CSMD3*	*FGFR1*	*IRF4*	*MLL2*	*PAX8*	*RNF2*	*TLR4*	
*BCL3*	*CTNNA1*	*FGFR2*	*IRS2*	*MLL3*	*PBRM1*	*RNF213*	*TLX1*	
*BCL6*	*CTNNB1*	*FGFR3*	*ITGA10*	*MLLT10*	*PBX1*	*ROS1*	*TNFAIP3*	
*BCL9*	*CYLD*	*FGFR4*	*ITGA9*	*MMP2*	*PDE4DIP*	*RPS6KA2*	*TNFRSF14*	
*BCR*	*CYP2C19*	*FH*	*ITGB2*	*MN1*	*PDGFB*	*RRM1*	*TNK2*	
*BIRC2*	*CYP2D6*	*FLCN*	*ITGB3*	*MPL*	*PDGFRA*	*RUNX1*	*TOP1*	
*BIRC3*	*DAXX*	*FLI1*	*JAK1*	*MRE11A*	*PDGFRB*	*RUNX1T1*	*TP53*	
*BIRC5*	*DCC*	*FLT1*	*JAK2*	*MSH2*	*PER1*	*SAMD9*	*TPR*	
*BLM*	*DDB2*	*FLT3*	*JAK3*	*MSH6*	*PGAP3*	*SBDS*	*TRIM24*	
*BLNK*	*DDIT3*	*FLT4*	*JUN*	*MTOR*	*PHOX2B*	*SDHA*	*TRIM33*	
*BMPR1A*	*DDR2*	*FN1*	*KAT6A*	*MTR*	*PIK3C2B*	*SDHB*	*TRIP11*	
*BRAF*	*DEK*	*FOXL2*	*KAT6B*	*MTRR*	*PIK3CA*	*SDHC*	*TRRAP*	
*BRD3*	*DICER1*	*FOXO1*	*KDM5C*	*MUC1*	*PIK3CB*	*SDHD*	*TSC1*	
*BRIP1*	*DLC1*	*FOXO3*	*KDM6A*	*MUTYH*	*PIK3CD*	*SEPT9*	*TSC2*	
*BTK*	*DNMT3A*	*FOXP1*	*KDR*	*MYB*	*PIK3CG*	*SETD2*	*TSHR*	
*BUB1B*	*DPYD*	*FOXP4*	*KEAP1*	*MYC*	*PIK3R1*	*SF3B1*	*UBR5*	
*CARD11*	*DST*	*FZR1*	*KIT*	*MYCL1*	*PIK3R2*	*SGK1*	*UGT1A1*	

**Table 2:** Annotations for somatic mutations of *NBN* and *PAX8*

Gene	Position	Exon	Coding DNA	Amino acid	COSMIC ID	SIFT	Polyphen2 HDIV	Mutation Taster	GQ	DP	AF
*NBN*	Chr8:90996774	1	c.16C>T	p.P6S	1102345	tolerated (0.13)	benign (0.429)	disease causing	58	122	0.11
*PAX8*	Chr2:114004376	3	c.146G>A	p.R49H	-	damaging (0)	Probably damaging (0.999)	disease causing	114	179	0.14

AF, allele frequency based on flow evaluator observation counts; DP, total read depth at the locus; GQ, genotype quality.

### Functional analysis of *PAX8*, p.R49H

**Figure 4:**
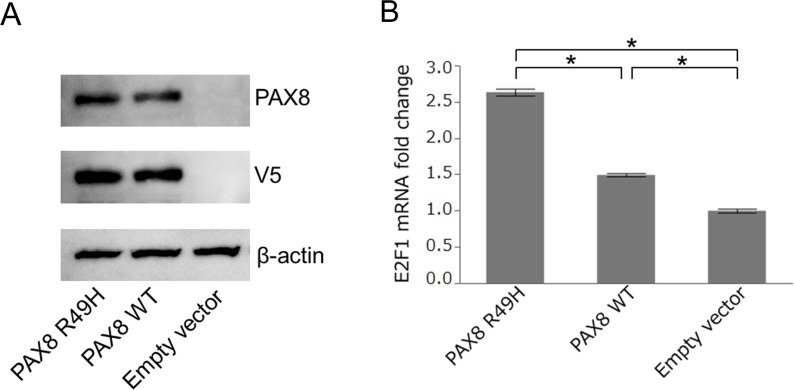
Transactivation of *E2F1* by *PAX8* **(A)** Immunoblot of 293T cells transfected with the *PAX8*^R49H^-V5-His (PAX8 R49H), *PAX8*^wild-type^-V5-His (PAX8 WT), and pcDNA3.1/V5-His vector (Empty vector) probed with antibodies of anti-PAX8, anti-V5, and anti-beta actin. **(B)** The relative expression of *E2F1* in each transfected 293T cells measured by the quantitative real-time PCR assay. The expression of *E2F1* was normalized to the expression of *GAPDH* and analyzed by means of the 2^−ΔΔCT^ method. An asterisk indicated P < 0.001.

To determine a functional phenotype of the *PAX8*^R49H^, we compared transactivation activities between the wild type *PAX8* and the mutant *PAX8*, i.e., *PAX8*^R49H^, toward *E2F1* that was known to be a transcriptional target of paired box 8 (PAX8) [9]. We constructed expression vectors harboring the wild type *PAX8* or the mutant *PAX8*, and transfected them into 293T cells. The transfection induced an equivalent level of exogenous expression of encoded proteins as indicated by immunoblots in Figure 4A. Then, we measured transcriptions of *E2F1* in the cells by the quantitative real time PCR assay. The result showed that the mutant *PAX8* induced a significantly increased level of transcription of *E2F1* compared to the wild-type *PAX8* as shown in Figure 4B (P < 0.001). These results indicate that the *PAX8*^R49H^ may exhibit a gain-of-function phenotype compared to the wild-type *PAX8*.

## DISCUSSION

Taking a molecular pathologic approach, we examined a peculiar case of gastric intramucosal adenocarcinoma with LN metastasis. The primary tumor seemed to fulfill the criteria for the absolute indication of ER; however, because of the LN metastasis, we performed a surgical resection with lymphadenectomy. In pathological examination, the primary tumor was accompanied by dense lymphatic vessels, and I-LVD proved to be particularly high. This finding suggests that the elevated level of lymphangiogenesis accompanying the adenocarcinoma could have increased the chances of primary cancer cells intravasating lymphatic vessels. LVD is known to be associated with LN metastasis in various human cancers [10]. As for gastric cancer, although differences in the pathological roles of I-LVD and P-LVD are still controversial, several reports showed that I-LVD was higher in tumors associated with LN metastasis [5, 11, 12]. To the best of our knowledge, our case is the first to show that high I-LVD may facilitate LN metastasis even in a case of an intramucosal gastric carcinoma that seemed to meet the criteria for the absolute indication of ER. In the present case, the gastric cancer and metastatic LN were surgically resected because the swollen LN detected by CT was proven to be a metastasis by laparoscopic biopsy before surgery. This demonstrates that careful evaluation of LN state using CT is important, as was previously reported [4]. When LN metastasis is not identifiable on CT, I-LVD could potentially be measured to assess the risk of metastasis after ER because I-LVD can be evaluated in the ER specimen.

Next, we investigated the coding exons of 412 cancer-associated genes by next-generation sequencing. As the primary cancer was uncommonly multicentric localized adjacent to each other, which suggests the possibility of a pre-existing mutational accumulation in the atrophic gastric mucosa due to a history of *H. pylori* infection as previously reported [13], we avoided using noncancerous mucosa as a normal control sample for this analysis. The results indicated that the present prometastatic intramucosal gastric cancer with extraordinary LN metastasis did not harbor any common mutations for gastric cancer such as *TP53*, *ARID1A*, *PIK3CA*, *CDH1*, *SMAD4*, *APC*, *KRAS*, or *RHOA* or its regulatory molecules, *AKAP13* and *DLC1*. Instead, the present case harbored somatic mutations in the LN metastasis: *NBN,* p.P6S, and *PAX8,* p.R49H. The identification of only a few mutated genes could be explained by the early stage of the cancer. Molecular pathologic information of such an early-stage cancer with an aberrant prometastatic nature can be a valuable source to help elucidate the mechanism of metastasis because analyzing such cases may lead to the identification of molecular alterations associated with metastasis in a small number of mutated genes.

*NBN* encodes nibrin (NBN), a member of the MRE11/RAD50 double-strand break repair complex [14]. A truncating mutation in *NBN* causes a defective response to DNA double-strand breaks, which results in an unstable genome and a predisposition to malignancies [15]. The mutated P6 residue of *NBN* is not located within any functional domains. However, the P6S mutation of *NBN* was identified in a patient with uterine corpus endometrioid carcinoma (COSM1102345 in the COSMIC database, http://cancer.sanger.ac.uk/cosmic). *NBN*, p.K653fs is reported to be identified in peritoneal metastasis of gastric cancer by whole-exome sequencing elsewhere [16]. Although functional impacts of *NBN*^P6S^ are not known, the mutation could cause genomic instability or copy number variations.

*PAX8* encodes paired box 8 (PAX8), a transcription factor required for the formation of thyroxine-producing follicular cells, of endodermal origin [17]. *PAX8*^R49C^ has been identified as a somatic mutation in gastric cancer according to the COSMIC database (COSM4084322); however, *PAX8*^R49H^ has not been reported. The R49 residue of *PAX8* is located within the paired box domain (Figure 3B), which may be the reason that *PAX8*^R49H^ was predicted to be functionally damaging by some prediction programs, namely Polyphen-2 (http://genetics.bwh.harvard.edu/pph2/), SIFT (http://sift.jcvi.org/), and MutationTaster (http://www.mutationtaster.org/). Missense mutations within the paired box domain, such as p.Q40P, p.S54G, p.C57Y, p.L62R, are known to cause congenital hypothyroidism, thyroid hypoplasia and aplasia, and/or kidney agenesis due to the loss of its transactivation effect [18–21]. *PAX8* is expressed highly in the thyroid and kidney as well as slightly but evidently in gastric mucosa and gastric cancer (GEO10420251 and GEO95672775 in Gene Expression Omnibus Profiles, https://www.ncbi.nlm.nih.gov/geoprofiles). *PAX8* is reported to be expressed in metastatic non-small cell lung cancers and to promote cell migration via interaction with MET and RON [22].

By our experiments testing a transactivating function of *PAX8* toward *E2F1*, we found that the *PAX8*^R49H^ was able to show a gain-of-function phenotype in transactivation of *E2F1* compared to the wild type *PAX8*. E2F transcription factor 1 (E2F1) encoded by *E2F1* is well known for its tumor suppressive role in conjunction with retinoblastoma protein 1 (RB1), however, it is also known to play some promoting roles in cancer as reported to promote an epithelial-mesenchymal transition (EMT) by transactivating *FOXL2* in gastric cancer cells, which may be associated with increased LN metastasis in patients with gastric cancer [23]. Thus, our result and these compelling evidences suggest that *PAX8*^R49H^ could have promoted EMT and subsequent LN metastasis via E2F1 in the present case.

In the present case, the somatic mutations were identified only in the metastatic LN tissue and not in the primary tumor. To explain this crucial observation, one can argue that the LN metastasis was not derived from the gastric adenocarcinoma but from elsewhere in other organs. However, we determined that the LN tumor was likely to be a metastasis of the intramucosal gastric carcinoma, which was cured by the surgical resection, through the following evidence: identical histology between the gastric tumor and the metastatic LN tumor, anatomical location of the metastatic LN, and the concerted decrease of elevated tumor markers. Moreover, the patient has not manifested any other malignant neoplasms for more than 3 years after surgery and no adjuvant chemotherapy. Alternatively, the failure of finding mutations in the intramucosal gastric carcinoma could be due to tumor heterogeneity, i.e., a small number of prometastatic clones in the primary tumor. A previous report indicated that a lethal metastatic clone of prostate cancer was derived from only a single small lesion in 36 sectioned blocks of the primary cancer [24]. Thus, it is likely that cells with a metastatic ability may have existed as just a tiny fraction in the primary tumor, which could not be detected. On the other hand, we could not exclude the possibility that the mutations occurred after metastasizing to the LN. Postmetastatic mutations could give advantages of survival and growth of cells in the metastatic site. Fractions of mutated calls were 11% and 14% in *NBN* and *PAX8*, respectively. This relatively minor fraction of mutated alleles may be due to 1) heterogeneity of cancer cells, 2) wild cancer cells derived from the collective dissemination of tumor clusters [25], or 3) normal lymphocytes with density drastically higher than that of the cancer cells, as shown in Figure 1C, meaning the ratio of cancer-derived DNA should be substantially lowered by contamination of even a small volume of peripheral lymphocytes. This could encourage the assumption that these mutations contributed to facilitation and/or development of LN metastasis, since they are more selected in the metastatic LN.

In conclusion, intramucosal gastric carcinoma that seemed to fulfill the criteria for the absolute indication of ER had a LN metastasis and thus was resected surgically. Increased lymphangiogenesis was observed in the primary tumor. Moreover, somatic mutations of *NBN*, p.P6S, and *PAX8*, p.R49H were observed in the metastatic tumor. The *PAX8*^R49H^ showed a gain-of-function phenotype in transactivation of *E2F1*. These findings may serve not only to develop biomarkers and/or molecular therapeutic targets but also to revise current recommendation for ER resection of early gastric cancer.

## MATERIALS AND METHODS

### Ethics and informed consent

This study was approved by the ethical committee of Tokyo Women's Medical University (protocol #272). Written informed consent was obtained from the patient for research and publication.

### Quantitative analysis of lymphatic vessel density

FFPE tissue sections were immunohistochemically stained by using anti-D2-40 antibody (Covance Antibody Products, San Diego, CA, USA) and Autostainer Link 48 (Dako, Glostrup, Denmark). Quantitative analysis of lymphatic vessel density (LVD) was performed by counting the D2-40 stained lymphatic vessels according to Pak et al [5]. Intratumoral (I)-LVD, Peritumoral (P)-LVD, and LVD in the normal control tissues (C-LVD) were counted.

### Panel design for the next-generation sequencing

The Ion AmpliSeq™ Comprehensive Cancer Panel covering all coding exons of 409 cancer-associated genes and an Ion AmpliSeq™ Custom DNA Panel covering all coding exons of *RHOA* and its regulatory molecules, *AKAP13* and *DLC1,* were used. In total, all coding exons of 412 genes were examined (Table 1).

### Tissue dissection and DNA preparation

FFPE tissue samples from the primary gastric tumor, the metastatic LN tumor, and a normal portion of the stomach (submucosa or deeper area) were used for genetic analysis. Areas of adenocarcinoma found by microscopic observation were manually dissected. DNA was prepared with a QIAamp DNA FFPE Tissue Kit (Qiagen, Hilden, Germany). DNA from the metastatic LN tumor and normal gastric tissue were analyzed by next-generation sequencing. For the primary cancer, since each lesion was small, we mixed the DNA together and only analyzed it by Sanger sequencing.

### Next-generation sequencing

Sequencing libraries were prepared using Ion AmpliSeq™ Library Kit 2.0 (Thermo Fisher Scientific) according to the manufacturer's instruction. The quantity of DNA amplicons was evaluated using a High Sensitivity DNA kit (Agilent Technologies, Waldbronn, Germany). Sequencing was performed using an Ion Proton™ System (Thermo Fisher Scientific) according to the manufacturer's instructions.

### Variant calling and annotation

Data analyses were performed using the Ion Torrent Suite Software (version 5.0.3). After base calling, the reads were aligned against the reference human genome (hg19) using the TMAP algorithm within the Torrent Suite. Variants with Genome quality > 50 and an allele frequency > 10% were considered. For further single nucleotide polymorphism (SNP) analysis, only non-synonymous nucleotide exchanges were considered. SNPs reported to be > 1% in 1000G, ExAC or ESP6500si were dismissed. SNPs detected only in tumor tissues were counted. All somatic variations annotated were validated by Sanger sequencing.

### Sanger sequencing

Genomic portions of somatic mutations were amplified by using paired primers of 5′-GGTTACGCGGTTGCACGTCG-3′ and 5′-TCTGCCC TTACCTCCTGCCG-3′ for *NBN* and 5′-CTTTGTGAA TGGCAGACCTC-3′ and 5′-AAGGATCTTGCTGACGCA GC-3′ for *PAX8*. The amplified products were analyzed by Sanger sequencing, as described previously [26].

### Cell culture

The human embryonic kidney 293T was obtained from the European Collection of Authenticated Cell Cultures (ECACC 12022001). The cells were cultured using Dulbecco's Modified Eagle's Medium (Sigma, St. Louis, MO, USA) with 10% fetal bovine serum in a humidified incubator at 37°C with 5% CO2.

### Plasmid vectors

The wild-type *PAX8* cDNA was amplified from a fetal kidney cDNA library (Agilent Technologies) by PCR using the KOD Plus DNA polymerase system (TOYOBO, Osaka, Japan). The paired primers used were as follows: forward 5′-TTTAAGCTT/CCCCGGCGATGCCTCACAAC-3′, and reverse 5′-TTTGAATTC/CAGATGGTCAAAGGCCGTGGC-3′. The amplified product was separated by agarose gel electrophoresis. A band corresponding to an equivalent molecular weight of *PAX8* (NM_003466.3) was extracted, purified, and cloned into the pcDNA3.1/V5-His vector (Invitrogen, San Diego, CA, USA) at the HindIII and EcoRI sites to generate the wild-type PAX8-V5-His vector. The mutant *PAX8* (p.R49H)-V5-His vector was generated by means of a site-directed mutagenesis technique using QuikChange II Site-Directed Mutagenesis Kit (Agilent Technologies) with following primers: 5′- GACGCGGAGCTGGTGAGAGATGTCGCAG-3′ and 5′-CTGCGACATCTCTCACCAGCTCCGCGTC-3′ according to the manufacture's instruction. Nucleotide sequences of the created plasmid vectors were confirmed by Sanger sequencing.

### Cell transfection

293T cells were seeded at a concentration of 5×10^5^ cells/well in 6-well plates 24 h before transfection. Transfection of each created plasmid vectors or the control pcDNA3.1/V5-His vector was performed using Lipofectamine 2000 Transfection Reagent (Thermo Fisher Scientific) according to the manufacturer's instruction. 24 h after transfection, cells were collected and proceeded to following immunoblotting and the quantitative real-time PCR assay. The experiment was performed twice in duplicate.

### Immunoblotting

Collected cells were lysed in modified RIPA buffer containing 1×complete mini protease inhibitor cocktail (Sigma) and 1×PhosSTOP phosphatase inhibitor cocktail (Sigma). Cell extracts containing equal amounts of proteins were boiled in loading buffer, applied to 10-20% polyacrylamide gradient gel, and separated by electrophoresis. Then the proteins were blotted onto a polyvinylidene difluoride membrane (ATTO, Tokyo, Japan). After blocking using the ECL Blocking Agent (Amersham Biosciences, Buckinghamshire, UK) for 1 h, the membrane was incubated with primary antibodies overnight. Primary antibodies used were the mouse monoclonal anti-PAX8 antibody (1:200 dilution) (Santa Cruz Biotechnology, Dallas, TX, USA), the mouse monoclonal anti-V5 antibody (1:5000 dilution) (Thermo Fisher Scientific), and the mouse monoclonal anti-β-actin antibody (1:1000 dilution) (Sigma). The membrane was incubated with a secondary antibody for 1 h. The secondary antibody used was horseradish peroxidase-conjugated anti-mouse immunoglobulin antibody (1:10000 dilution) (GE Healthcare, Buckinghamshire, UK). Signals were visualized using the ECL Prime Western Blotting Detection Reagent (Amersham Biosciences) and LAS 4000 Mini system (Fujifilm, Tokyo, Japan).

### Quantitative real-time PCR assay

Total RNA was isolated from collected cells using RNeasy Mini kit (Qiagen, Hilden, Germany). cDNA synthesis was performed using High-Capacity cDNA Reverse Transcription Kit (Thermo Fisher Scientific). Pre-designed primer/probe sets for human *PAX8* (Hs00247586_m1) and *E2F1* (Hs00153451_m1) in TaqMan gene expression assay system (Thermo Fisher Scientific) were used in the quantitative real-time PCR assay. GAPDH was used as an endogenous control. The analyses were performed by means of the 2^−ΔΔCT^ method [27] upon the 7500 Real-Time PCR system (Thermo Fisher Scientific) according to the manufacture's instruction. The experiment was performed twice in triplicate.

### Statistical analyses

Continuous data are described as the mean and standard deviation and were compared using Tukey's method.

## SUPPLEMENTARY MATERIALS FIGURE



## Abbreviations

CA19-9: carbohydrate antigen 19-9
CEA: carcinoembryonic antigen
C-LVD: Lymphatic vessel density in the normal control tissues
CT: computed tomography
EMT: epithelial-mesenchymal transition
ER: endoscopic resection
FFPE: Formalin-fixed and paraffin-embedded
H&E: hematoxylin and eosin
I-LVD: Intratumoral-lymphatic vessel density
LN: lymph node
LVD: lymphatic vessel density
NBN: nibrin
PAX8: paired box 8
PCR: polymerase chain reaction
P-LVD: Peritumoral lymphatic vessel density
RB1: retinoblastoma protein 1.
