# Supplementary material for: Somatic mutations and increased lymphangiogenesis observed in a rare case of intramucosal gastric carcinoma with lymph node metastasis
Source: Oncotarget. 2018 Jan 22;9(12):10808–17. doi: 10.18632/oncotarget.24289 (PMC5828222; doi:10.18632/oncotarget.24289)
Supplement: Supplementary file 1 [file oncotarget-09-10808-s001.pdf]

Somatic mutations and increased lymphangiogenesis observed in a rare case of intramucosal gastric carcinoma with lymph node metastasis

SUPPLEMENTARY MATERIALS

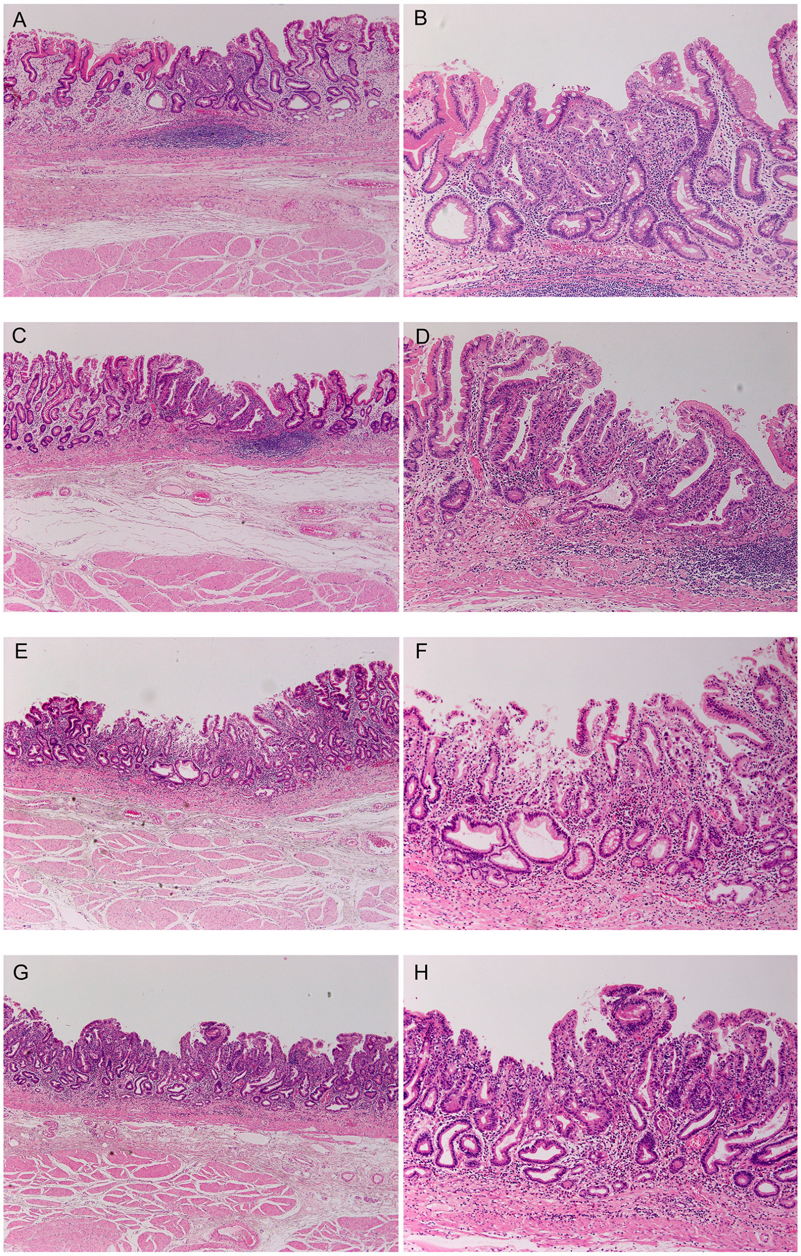

Supplementary Figure 1: Pathological findings of each primary lesion of a multicentric gastric cancer. Each lesion consisted of well-differentiated tubular adenocarcinoma, was confined within the mucosal layer, and was 1-4 mm in diameter without ulceration. Lamina muscularis mucosae between the lesions were conserved, which suggests that segregation of a single lesion into 4 lesions was unlikely. Original magnification: ×40 in panels **(A, C, E, and G)**; and ×100 in **(B, D, F, and H)**.
